# Ethnicity should be included as a risk factor for coronary artery calcium score

**DOI:** 10.1186/s12933-022-01587-5

**Published:** 2022-08-08

**Authors:** Ibrahim Al-Sawalha, Dalia Alzoubi

**Affiliations:** Faculty of Medicine, Jordan University of Science and Technology, Irbid, Jordan

**Keywords:** Coronary artery calcium score, Ethnicity, Myocardial infarction

## Abstract

Coronary artery calcium score (CACs) is a measurement of calcification in coronary arteries using a computed tomography scan. It has a predictive role for future myocardial infarction thus it aids in cardiovascular risk assessment and physician decision making. Multiple risk factors have been attributed to coronary artery calcification including age, male gender and ethnicity. Herein, we report our concerns regarding neglecting ethnicity within participants demographics and data analysis, which may affect the findings of the study.

We read with great interest the article by Shi et al. [1] as this paper investigated the association between LDL-C status and coronary artery calcium score (CACs) in statin-treated diabetic patients. This study absolutely contributes to the current literature on diabetic population and cardiovascular risk assessment. Although the study methodology and result reporting are well conducted, we have concerns regarding the risk factors included within participants’ demographics.

Taking in consideration CACs as one of the important variables investigated in this study, it is questionable why the authors did not take the ethnicity of participants into account. The Multi-Ethnic Study of Atherosclerosis (MESA) found significant difference between CAC score and ethnicity [2]. MESA investigated four ethnicities (white, black, Hispanic and Chinese), and concluded that coronary calcification with an Agatston score > 0 was greatest among the white race and least in the black race. Furthermore, ethnicities have shown influence on other cardiovascular risk factors in diabetic patients. It was reported that diabetic patients with hypertriglyceridemia were more likely to be white and report Hispanic/Latino ethnicities than other patients with normal triglyceride levels [3]. In addition, epicardial adipose tissue volume was found to be higher in diabetic patients of Caucasian ethnicity and it was independently associated with subclinical coronary atherosclerosis [4].

Gender differences in various ethnicities were also reported to affect lipoproteins impact on coronary artery disease (CAD) risk. It was found that lipoproteins, especially HDL, seem to reduce CAD risk in African American women and not in other women [5]. Finally, a study by Nasir et al. [6] concluded that ethnicities significantly influence the baseline CAC score and the prognostic value of CAC score on survival.

Neglecting ethnicities of participants affects multivariate analysis models used in this study. Since they are not adjusted for ethnicity, this could alter the findings of the study. Therefore, the use of ethnicity as a risk factor would have improved the analysis models, strengthen the study findings and made them more valid.

## Data Availability

Not applicable.
